# SPHK1‐induced autophagy in peritoneal mesothelial cell enhances gastric cancer peritoneal dissemination

**DOI:** 10.1002/cam4.2041

**Published:** 2019-02-21

**Authors:** Songcheng Yin, Zhifeng Miao, Yuen Tan, Pengliang Wang, Xiaoyu Xu, Chao Zhang, Wenbin Hou, Jinyu Huang, Huimian Xu

**Affiliations:** ^1^ Department of Surgical Oncology First Affiliated Hospital of China Medical University Shenyang China; ^2^ Key Laboratory of Gastric Cancer Molecular Pathology of Liaoning Province Heping District Shenyang China; ^3^ Department of Gynecology The Seventh Affiliated Hospital of Sun Yat‐sen University Shenzhen China

**Keywords:** autophagy, gastric cancer peritoneal dissemination, mesothelial cell, SPHK1

## Abstract

Gastric cancer peritoneal dissemination (GCPD) has been recognized as the most common form of metastasis in advanced gastric cancer (GC), and the survival is pessimistic. The injury of mesothelial cells plays an important role in GCPD. However, its molecular mechanism is not entirely clear. Here, we focused on the sphingosine kinase 1 (SPHK1) in human peritoneal mesothelial cells (HPMCs) which regulates HPMCs autophagy in GCPD progression. Initially, we analyzed SPHK1 expression immunohistochemically in 120 GC peritoneal tissues, and found high SPHK1 expression to be significantly associated with LC3B expression and peritoneal recurrence, leading to poor prognosis. Using a coculture system, we observed that GC cells promoted HPMCs autophagy and this process was inhibited by blocking TGF‐β1 secreted from GC cells. Autophagic HPMCs induced adhesion and invasion of GC cells. We also confirmed that knockdown of SPHK1 expression in HPMCs inhibited TGF‐β1‐induced autophagy. In addition, SPHK1‐driven autophagy of HPMCs accelerated GC cells occurrence of GCPD in vitro and in vivo. Moreover, we explored the relationship between autophagy and fibrosis in HPMCs, observing that overexpression of SPHK1 induced HPMCs fibrosis, while the inhibition of autophagy weakened HPMCs fibrosis. Taken together, our results provided new insights for understanding the mechanisms of GCPD and established SPHK1 as a novel target for GCPD.

## 1 INTRODUCTION

Gastric cancer (GC) remains the fifth most frequent cancer and the third leading cause of cancer death worldwide in 2018.^1^ Gastrectomy‐based perioperative or postoperative adjuvant therapies have been applied, but prognosis remains unsatisfactory.^2^ Peritoneal dissemination (PD) is the most common cause of tumor progression in advanced GC, and the median survival of patients is only 4‐6 months.^3^, ^4^ However, the mechanisms underlying PD are not entirely clear. Furthermore, there is a lack of accurate diagnostic biomarkers and excellent therapeutic targets for PD.

Gastric cancer peritoneal dissemination (GCPD) is the result of interactions between tumor cells and the peritoneal microenvironment. Our previous studies clarified that transforming growth factor‐beta 1 (TGF‐β1) from GC cells stimulated human peritoneal mesothelial cell (HPMC) fibrogenesis.^5^, ^6^, ^7^ HPMC fibrosis, as a critical mediator of GCPD, promoted tumor cell adhesion and invasion.^7^

Recently, TGF‐β1 has also been reported to regulate autophagy, a process of bulk degradation of intracellular components through the formation of autophagosomes and degradation by lysosomes.^8^ Autophagy is critical for the homeostasis for normal proliferation and differentiation, and it is also an adaptive response to maintain cellular viability after exposure to stressful stimuli.^9^ Interestingly, it has been demonstrated that TGF‐β1 differentially regulates autophagy; specifically, the growth factor promotes autophagy in vascular endothelial cells^10^ and tubular epithelial kidney cells^11^, but inhibits this process in fibroblasts from patients with idiopathic pulmonary fibrosis.^12^ However, the autophagy level of HPMCs, especially in the GCPD microenvironment, remains largely unclear.

Sphingosine kinase 1 (SPHK1) catalyzes the phosphorylation of sphingosine to sphingosine 1‐phosphate.^13^ SPHK1 is widely involved in cell growth, proliferation, and antiapoptosis. In particular, SPHK1 plays an oncogenic role in promoting survival and invasion in some tumors.^14^ High SPHK1 expression promoted breast cancer cell proliferation and invasion, which were associated with poor overall survival.^15^ Additionally, SPHK1 participated in cisplatin and docetaxel resistance in gastroesophageal cancer.^16^ However, little is known about the role of SPHK1 in tumor stroma cells. Emerging evidence has implicated SPHK1 in cellular autophagy in pathological conditions.^17^ Nevertheless, the precise roles of SPHK1 in HPMCs autophagy, in addition to the regulatory mechanisms and the relationship of SPHK1 with GCPD, should be confirmed.

In the present study, we found that overexpression of SPHK1 in HPMCs was associated with LC3B expression (an autophagy protein marker), peritoneal recurrence, and poor overall survival. Using a coculture system, we observed that GC cells secreted TGF‐β1, which promoted HPMCs autophagy by regulating SPHK1. In addition, SPHK1‐driven autophagy accelerated the occurrence of GCPD. Moreover, we explored the relationship between autophagy and fibrosis in HPMCs, observing that the regulation of HPMCs fibrosis was partially induced by SPHK1‐induced autophagy.

## 2 MATERIALS AND METHODS

### 2.1 Patient tissue specimens

In total, 120 patients with GC who underwent radical surgery at the First Hospital of China Medical University between 2003 and 2010 were included in this study. Resected peritoneal tissues were fixed and embedded in paraffin. All patients were approved for study participation by the Ethics and Indications Committee of China Medical University. Written informed consent was provided for all patients. The TNM stage of the patients was restaged according to the eighth edition of the AJCC cancer staging manual.

### 2.2 Immunohistochemistry

Immunohistochemistry (IHC) staining was performed in line with the routine protocols.^18^, ^19^, ^20^ Sections were deparaffinized using xylene and hydrated through an ethanol gradient. Endogenous peroxidase activity was blocked by incubation in 3% H_2_O_2_ for 15 minutes, and antigen retrieval was conducted using 0.01 mol L^−1^ sodium citrate buffer (pH 6.0) for 3 minutes at high pressure. After blocking in 10% normal goat serum, sections were incubated with a primary antibody against SPHK1 (1:200, Abcam, Cambridge, MA, USA) LC3B (1:400, Abcam, Cambridge, MA, USA) at 4°C overnight. Sections were incubated with HRP‐conjugated secondary goat anti‐rabbit antibody at 37°C for 30 minutes and exposed to 3,3′‐diaminobenzidine. Subsequently, sections were counterstained with hematoxylin. Staining was judged by the percentage of positive cells and staining intensity. The percentage of positive cells was scored as follows: <5% (0), 5%‐25% (1), >25%‐50% (2), >50%‐75% (3), and >75% (4). The scoring system for staining intensity was as follows: negative (0), weak (1), moderate (2), and strong (3). The staining index was calculated by multiplying the score for the percentage of positive cells by that for staining intensity. An index of 6‐12 indicated high expression, whereas scores of 0‐4 represented low expression.

### 2.3 Cell lines and cell culture

A human peritoneal mesothelial cell (HPMC) line, which was established by Prof. Ronco,^21^ was kindly provided by Prof. You‐Ming Peng (Second Hospital of Zhongnan University, Changsha, China). HPMCs were cultured in RPMI 1640 medium containing 10% fetal bovine serum (FBS). Human GC cell lines MKN‐45, MKN‐28, SGC‐7901, MGC‐803, and BGC‐823 were purchased from the Cell Bank of Type Culture Collection of Chinese Academy of Sciences (Shanghai, China). Human GC cells were cultured in RPMI 1640 medium or DMEM supplemented with 10% FBS. All cell cultures were incubated continuously under 5% CO_2_ at 37°C. For the GC and HPMC coculture system, transwell chambers (0.4‐μm pores, Corning) separated by a polycarbonate membrane were used similar to the previous experiments performed in our laboratory.^6^, ^22^ GC cells (5.0 × 10^5^ cells) were seeded into the top chamber, and HPMCs (1.0 × 10^5^ cells) were placed in the bottom compartment. GC cells had no direct contact with HPMCs, but the soluble factors derived from the GC cell lines could reach HPMCs.

### 2.4 Transfection

TGF‐β1‐RNAi and SPHK1‐RNAi lentiviruses were constructed by GeneChem (Shanghai, China). GC cells or HPMCs were infected with lentiviral particles in the presence of polybrene according to the manufacturer's instructions. Infected cells were selected using 5 μg/ml puromycin (Sigma, USA). Transfection with Flag‐SPHK1 and negative‐control plasmids (GeneChem, Shanghai, China) was performed using Lipofectamine 3000.

### 2.5 Immunoblot analysis

Western blotting was performed as previously described.^18^ Briefly, cells were harvested, lysed, and centrifuged. Equal amounts of proteins were separated by SDS‐PAGE and transferred onto PVDF membranes (Millipore). The membranes were blocked in 5% nonfat milk and incubated with primary antibodies for TGF‐β1, SPHK1, E‐cadherin, N‐cadherin, α‐SMA, and GAPDH (Abcam) at 4°C overnight. After incubation in the corresponding secondary antibodies, membranes were incubated in the enhanced chemiluminescence solution (Thermo Fisher Scientific).

### 2.6 Enzyme‐linked immunosorbent assay (ELISA)

TGF‐β1 levels in the culture supernatant of GC cells were measured using the Human TGF‐β1 Immunoassay Kit (R&D Systems) as described previously.^23^

### 2.7 Confocal microscopy

Human peritoneal mesothelial cells were transfected with recombinant mRFP‐GFP‐LC3 adenovirus (Hanbio Biotechnology, Shanghai, China) as described previously.^24^ In green‐ and red‐merged images, autophagosomes are shown as yellow puncta, whereas autolysosomes are shown as red puncta. In addition, autophagic flux was detected using a scanning confocal microscope.

### 2.8 Transmission electron microscopy

As described previously,^25^ HPMCs were fixed with 2.5% glutaraldehyde in 0.1 mol L^−1^ sodium cacodylate buffer, stored at 4°C overnight, and postfixed with 1% OsO4 for 1.5 hours. After staining with 3% aqueous uranyl acetate and dehydration in a graded ethanol series, cells were embedded in Epon resin. Ultrathin sections were examined using a transmission electron microscope.

### 2.9 Adhesion and invasion assay

The adhesion and invasion of GC cells in the presence of HPMCs were determined as described previously.^22^ For the adhesion assay, HPMCs (precedingly cocultured with GC) were plated in 96‐well plates. GC cells were incubated with 5 μmol L^−1^ Calcein‐AM (Sigma, USA) and added to HPMCs, and incubated for 1 hour. Afterward, the plates were washed three times with PBS to remove nonadherent GC cells. The cell number was counted under a fluorescence microscope. For the invasion assay, HPMCs cocultured with GC cells were seeded into the upper chamber of 24‐well transwell inserts (8‐μm pore size; Corning) that were coated with 100 μL of Matrigel. When the HPMCs reached 90% confluence, 2 × 10^5^ GC cells were resuspended in 100 μL of serum‐free medium and added into the upper chamber. The lower chamber was filled with 600 μL of DMEM containing 10% FBS as a chemoattractant. After incubation for the indicated times, cells on the lower surface were fixed and stained with 4% trypan blue. The percentage of stained cells was calculated under a microscope using five different fields of view.

### 2.10 In vivo GCPD assay

A xenograft model of cancer cells mixed with stromal cells was established as previously reported.^26^ Five‐week‐old male BALB/c nude mice (Beijing Vital River, China) were used. SGC‐7901 cells (2 × 10^6^) were mixed with HPMCs (5 × 10^5^) and implanted into the abdominal cavities of mice (n = 10). Mice were killed 6 weeks after implantation. Then, the locations of macroscopic tumors were recorded, and the tumor nodules were removed and weighed. All animal experimental procedures were approved by the Animal Research Committee of China Medical University.

### 2.11 Statistical analysis

A two‐tailed Student's *t* test was used to calculate average differences between groups. The Kaplan‐Meier method was used to conduct survival curves. Associations between different variables and overall survival were performed with the Cox proportional hazards regression model; hazard ratios (HRs) and 95% CIs were reported. The correlation between SPHK1 expression and clinicopathological factors in GC was calculated using the chi‐squared test. All statistical analyses were conducted using SPSS 21.0, and *P *<* *0.05 was considered statistically significant.

## 3 RESULTS

### 3.1 SPHK1 upregulation in the peritoneum is correlated with LC3B expression, peritoneal recurrence, and poor survival in GC

**Figure 1:**
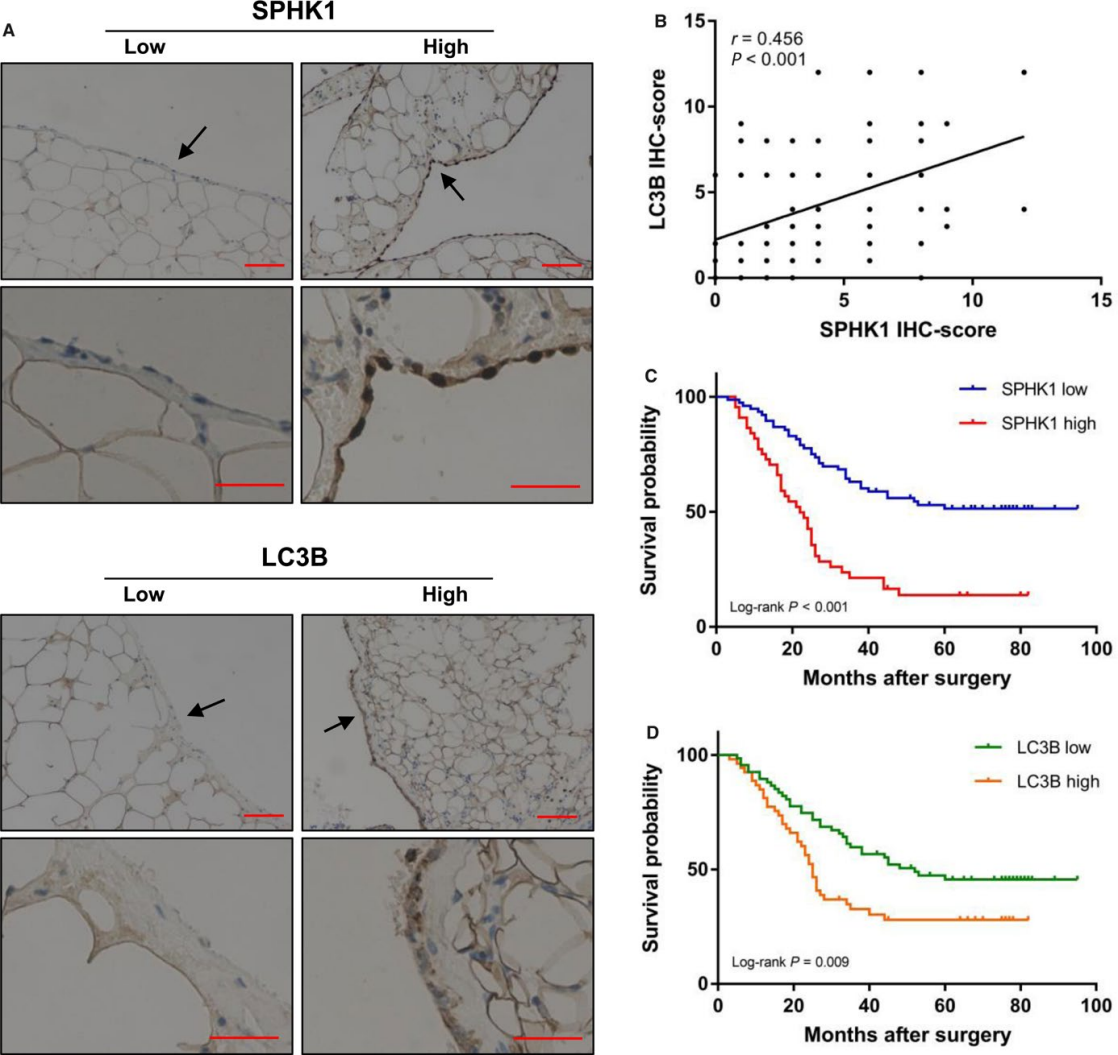
Upregulated SPHK1 in peritoneum is correlated with LC3B and poor survival in GC. (A) Representative immunohistochemistry (IHC) staining with SPHK1 and LC3B in GC peritoneum tissues. (B) Scatter plots showing the positive correlation between SPHK1 and LC3B IHC scores in peritoneum tissues. (C) Kaplan‐Meier survival curves based on SPHK1. (D) Kaplan‐Meier survival curves based on LC3B

We first investigated SPHK1 and LC3B expression in peritoneal tissues from 120 patients with GC. IHC analysis revealed that the SPHK1 and LC3B‐positive expression rates were 36.7 (44/120) and 45.0% (54/120), respectively (Figure 1A). High‐resolution images were included in the supplementary Figures [Link], [Link], [Link], [Link]. In addition, SPHK1 expression was positively correlated with LC3B expression in peritoneal tissues (Pearson's coefficient test, *r *=* *0.456; *P *<* *0.001; Figure 1B). We further analyzed the association of SPHK1 with clinicopathological characteristics and prognosis in these patients. High SPHK1 expression was significantly associated with larger tumor size, deeper depth of tumor invasion, lymph node metastasis, advanced TNM stage, high LC3B expression, and peritoneal recurrence (Table 1). Conversely, there was no correlation of SPHK1 expression with gender, age, and tumor differentiation. Kaplan‐Meier analysis illustrated that patients with high SPHK1 and LC3B expression had poor OS (*P*_SPHK1_ < 0.001, *P*_LC3B_ = 0.009, Figure 1C and D). After adjustment for potential confounding factors, multivariate Cox regression analysis identified SPHK1 upregulation as an independent factor for OS (*P *=* *0.031, Table 2).

**Table 1:** Clinicopathological characteristics and staining patterns of SPHK1 in gastric cancer

Variables	SPHK1 expression		*P‐*value
	High (44)	Low (76)	
Age			0.463
<65	26	50	
≥65	18	26	
Sex			0.627
Male	14	21	
Female	30	55	
Tumor size			0.046
<5 (cm)	16	42	
≥5 (cm)	28	34	
Differentiation			0.690
Well/moderate	19	30	
Poor	25	46	
Depth of tumor invasion			< 0.001
pT1‐3	10	45	
pT4	34	31	
Lymph node metastasis			0.032
Absent	9	30	
Present	35	46	
pStage			0.005
I‐II	9	35	
III	35	41	
Peritoneal recurrence			<0.001
Absent	21	64	
Present	23	12	
LC3B expression			0.004
Low	17	50	
High	27	26	

**Table 2:** Univariate and multivariate Cox proportional hazards analyses on overall survival for gastric cancer patients

Parameters	Univariate analysis		Multivariate analysis	
	HR (95% CI)	*P*‐value	HR (95% CI)	*P*‐value
Age (≥65 years)	0.532 (0.335‐0.845)	0.007		
Gender (male)	0.827 (0.494‐1.383)	0.469		
Tumor size (≥5 cm)	1.474 (0.926‐2.345)	0.102		
Differentiation (poor)	0.804 (0.505‐1.280)	0.358		
Depth of tumor invasion (T1‐T4)	1.752 (1.351‐2.272)	<0.001	1.384 (1.028‐1.864)	0.032
Lymph node metastasis (+)	1.508 (1.272‐1.786)	<0.001	1.298 (1.067‐1.578)	0.009
Peritoneal recurrence (+)	4.240 (2.622‐6.857)	<0.001	2.600 (1.545‐4.375)	<0.001
High SPHK1 expression	3.114 (1.950‐4.975)	<0.001	1.826 (1.057‐3.155)	0.031
High LC3B expression	1.824 (1.147‐2.899)	0.011		

HR, hazard ratio; CI, confidence interval.

### 3.2 GC cell line SGC‐7901 upregulates SPHK1 expression in HPMCs and induced HPMC autophagy via TGF‐β1

**Figure 2:**
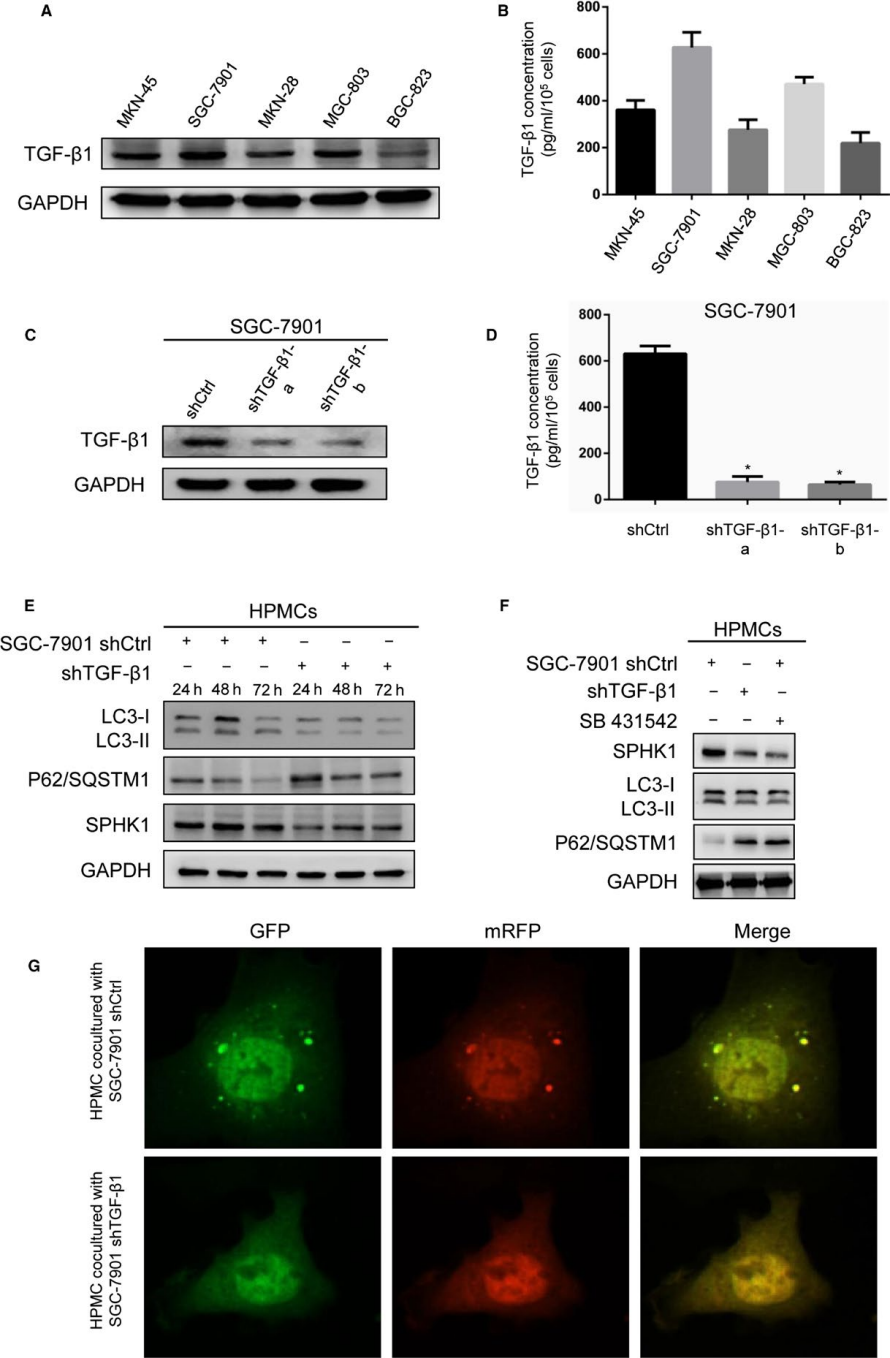
GC cell line SGC‐7901 upregulated HPMCs SPHK1 expression and induced HPMCs autophagy via TGF‐β1. (A) TGF‐β1 expression in five GC cell lines detected by western blotting. (B) TGF‐β1 levels in condition medium of five GC cell lines analyzed by ELISA. (C) Protein expression of TGF‐β1 after transfection with shTGF‐β1 lentivirus in SGC‐7901. (D) TGF‐β1 levels in condition medium of SGC‐7901 transfected with shRNA lentivirus. (E) Western blot showing the expression of LC3B, P62/SQSTM1, and SPHK1 in HPMCs cocultured with SGC‐7901 after 24, 48, and 72 hour. (F) The effect of TGF‐β1 receptor inhibitor SB431542 on LC3B, P62/SQSTM1, and SPHK1 expression in HPMCs. (G) Immunofluorescent micrographs demonstrating mRFP‐GFP‐LC3 fusion protein in HPMCs cocultured with SGC‐7901 after 48 hour. ^⁎^ *P* < 0.05

Considering that TGF‐β1 is an important paracrine protein, we first investigated TGF‐β1 levels in intracellular and in culture supernatants in GC cell lines via western blotting and ELISA. Consistently, SGC‐7901 cells presented highest levels of TGF‐β1 in intracellular and in culture medium (Figure 2A and B). Thus, SGC‐7901 cells were selected for use in the coculture model for subsequent experiments. Then, SGC‐7901 cells were transfected with shRNAs. TGF‐β1 expression was efficiently suppressed, as determined via western blotting and ELISA (Figure 2C and D).

To investigate the role of GC cells in the promotion of HPMC autophagy, we cocultured SGC‐7901‐shCtrl/shTGF‐β1 cells with HPMCs. After 72 hour of coculture, autophagy‐related protein expression in HPMCs was assayed via western blotting. We observed decreased LC3‐II expression and increased p62/SQSTM1 expression in SGC‐7901‐shTGF‐β1‐cocultured HPMCs compared with the expression in SGC‐7901‐shCtrl‐cocultured cells at 24, 48, and 72 hour (Figure 2E). Meanwhile, TGF‐β1 signaling blockade attenuated SPHK1 expression. Consistently, the TGF‐β1 receptor inhibitor SB431542 also inhibited SPHK1 expression and autophagy by upregulating p62/SQSTM1 and downregulating LC3‐II (Figure 2F). In addition, we detected autophagic vesicles via confocal microscopy. The number of yellow puncta were significantly decreased in the SGC‐7901‐shTGF‐β1 coculture group, which confirmed the reduction in the number of autophagosomes (Figure 2G).

### 3.3 HPMCs autophagy stimulates GC cell adhesion and invasion

**Figure 3:**
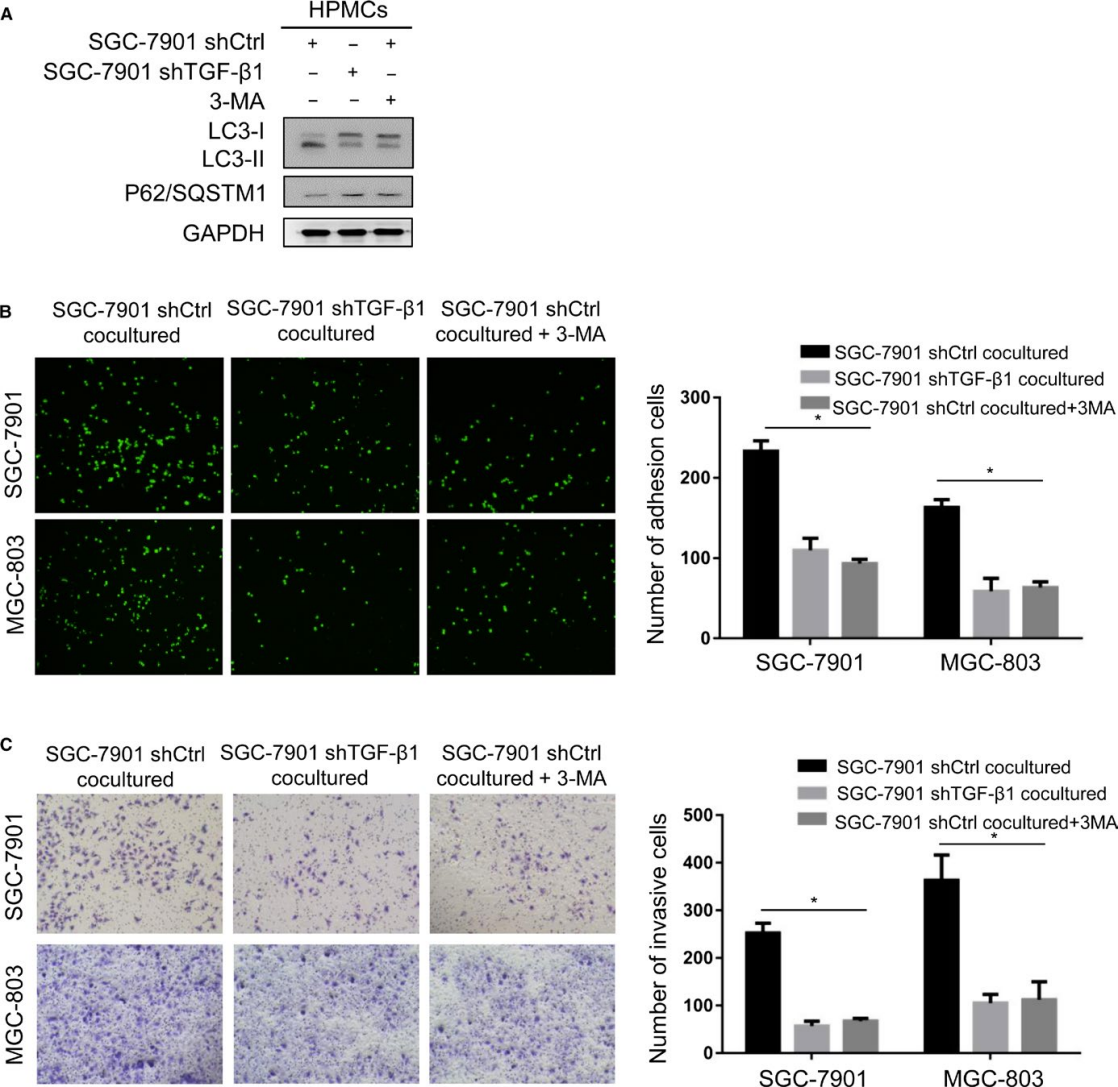
Autophagy of HPMCs stimulated the adhesion and invasion of GC cells. (A) HPMCs were cocultured with SGC‐7901 cells (shCtrl or shTGF‐β1) in the absence/presence of the autophagy inhibitor 3‐MA (5 mmol L^−1^). Western blotting analysis revealed that 3‐MA abrogated the HPMCs’ autophagic effect of TGF‐β1 from SGC‐7901 cells. Adhesion (B) and invasion (C) of SGC‐7901 and MGC‐803 cells to the HPMCs cocultured with SGC‐7901 (shCtrl or shTGF‐β1) cells in the absence/presence of 3‐MA. ^⁎^ *P* < 0.05

To determine the role of HPMCs autophagy in the development of GCPD, cocultured HPMCs were treated with the autophagy inhibitor 3‐methyladenine (3‐MA) at a concentration of 5 mmol L^−1^. Figure 3A shows that SGC‐7901‐induced autophagy was significantly reduced in 3‐MA‐treated cells. Then, we performed adhesion and invasion assays for GC cells in peritoneal coculture models with HPMCs. By fluorescently examining the numbers of SGC‐7901 and MGC‐803 cells adhering to HPMCs, we found that the attachment of GC cells was significantly decreased for SGC‐7901‐shTGF‐β1‐cocultured and 3‐MA‐treated HPMCs compared with the findings for SGC‐7901‐shCtrl‐cocultured cells (Figure 3B). In the GC cell‐HPMC invasion assays, the SGC‐7901‐shTGF‐β1‐cocultured and 3‐MA‐treated HPMC monolayer barriers were less vulnerable to damage by GC cells (Figure 3C). These results suggested that TGF‐β1‐induced HPMC autophagy promoted the adhesion and invasion of GC cells.

### 3.4 SPHK1 is required for TGF‐β1‐induced HPMCs autophagy and GCPD promotion

**Figure 4:**
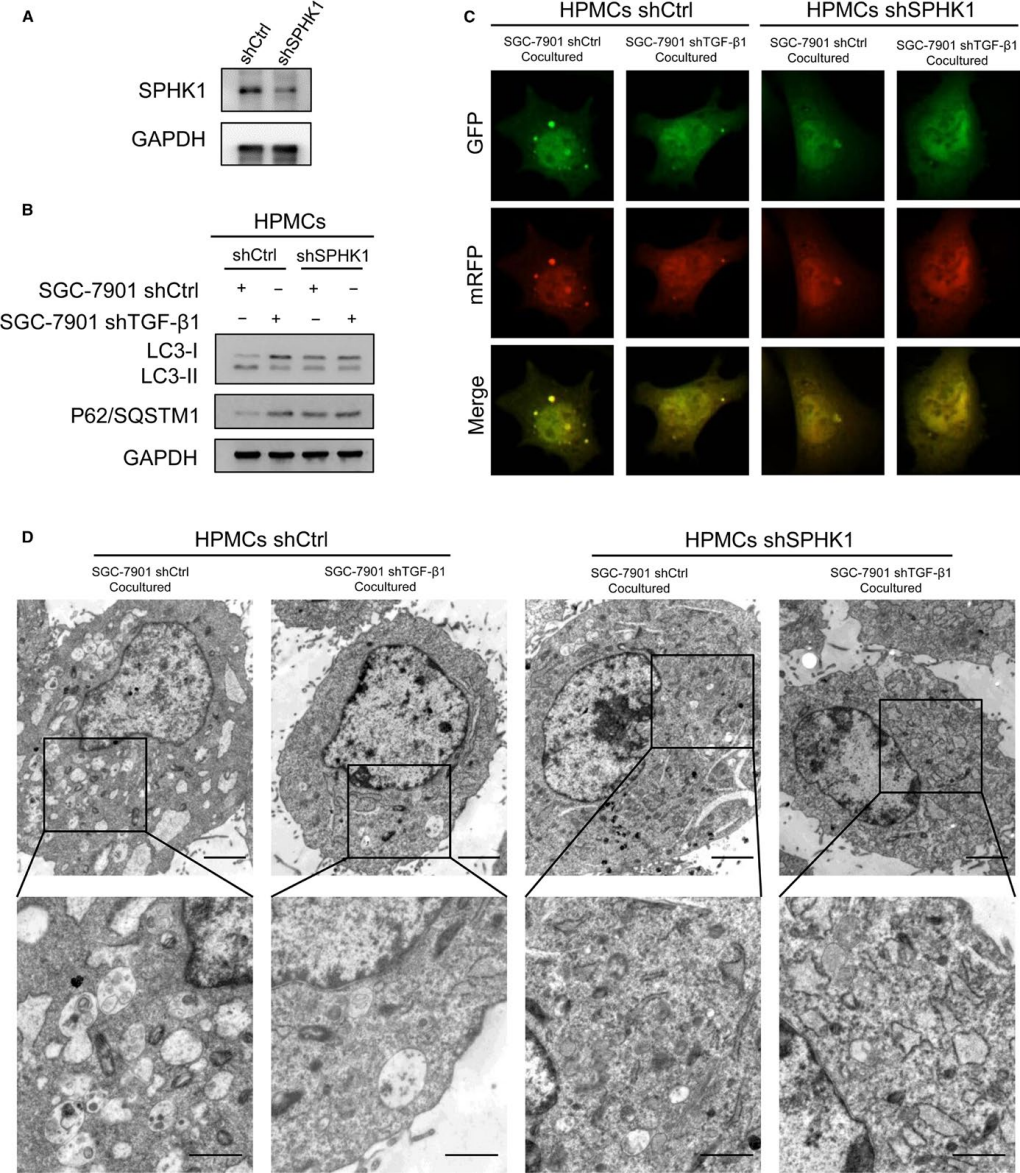
SPHK1 was required for TGF‐β1‐induced HPMC autophagy. (A) Western blotting detected SPHK1 expression after transfection with shSPHK1 lentivirus in HPMCs. (B‐D) HPMCs were transfected with shCtrl or shSPHK1 and cocultured with SGC‐7901 cells with existence or inhibition of TGF‐β1. (B) Western blotting analyzed the LC3B and P62/SQSTM1 expression in HPMCs. (C) Immunofluorescent micrographs and (D) transmission electron microscopy showed autophagosomes in HPMCs

Next, we tried to confirm the role of SPHK1 in TGF‐β1‐induced HPMC autophagy. We depleted SPHK1 expression in HPMCs using SPHK1 small interfering RNA (Figure 4A). Then, HPMCs were cocultured with SGC‐7901‐shCtrl and shTGF‐β1 cells. Western blotting revealed that SGC‐7901‐cocultured HPMCs transfected with SPHK1 shRNA exhibited decreased LC3 lipidation as well as p62/SQSTM1 degradation compared with the findings in shCtrl‐transfected cells (Figure 4B). Figure 4C obviously revealed that the numbers of autophagosomes and autophagolysosomes were decreased in SPHK1‐depleted HPMCs. As a gold standard for autophagosome detection, transmission electron microscopy revealed that autophagy‐induced TGF‐β1 paracrine signaling was significantly reduced by SPHK1 shRNA (Figure 4D).

**Figure 5:**
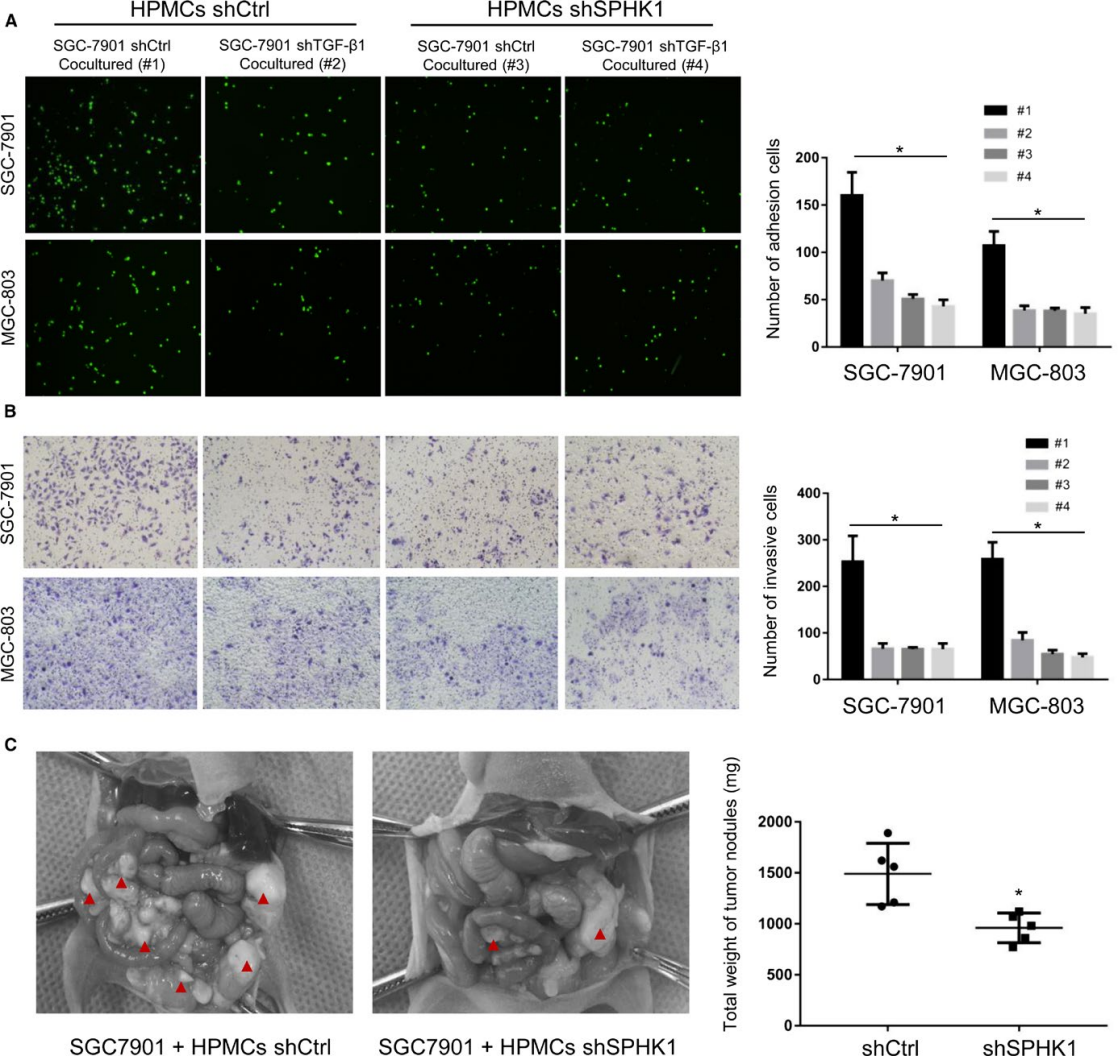
SPHK1 expression in HPMCs regulated GC cells adhesion, invasion, and GCPD. (A, B) HPMCs were transfected with shCtrl or shSPHK1 and cocultured with SGC‐7901 cells with existence or inhibition of TGF‐β1. Adhesion (A) and invasion (B) of SGC‐7901 and MGC‐803 cells to the HPMCs. (C) Representative tumor nodules in the abdominal cavity of nude mice that were intraperitoneally injected with SGC‐7901 cells admixed with shSPHK1 and shCtrl HPMCs, respectively. ^⁎^ *P* < 0.05

We further examined the effects of SPHK1 expression in HPMCs on the adhesion and invasion of GC cells. The attachment and invasion of SGC‐7901 and MGC‐803 cells to shSPHK1 HPMC monolayers was significantly inhibited (Figure 5A and B). To investigate the interactions between HPMCs and GC cells in GCPD in vivo, we created a mouse model. SGC‐7901 cells were injected intraperitoneally into BALB/c nude mice (n = 5 for each group) admixed with shSPHK1 or shCtrl HPMCs. The results demonstrated that SGC‐7901 cells coinjected with shSPHK1 HPMCs exhibited reduced macroscopic nodules during peritoneal cavity dissemination to the mesenterium, greater omentum, and parietal peritoneum (Figure 5C). In addition, the SGC‐7901/shSPHK1‐HPMC tumor weight was significantly lower than that of the matched tumors in which SPHK1 expression was not suppressed (Figure 5C).

### 3.5 SPHK1 regulates HPMCs fibrosis by promoting autophagy

**Figure 6:**
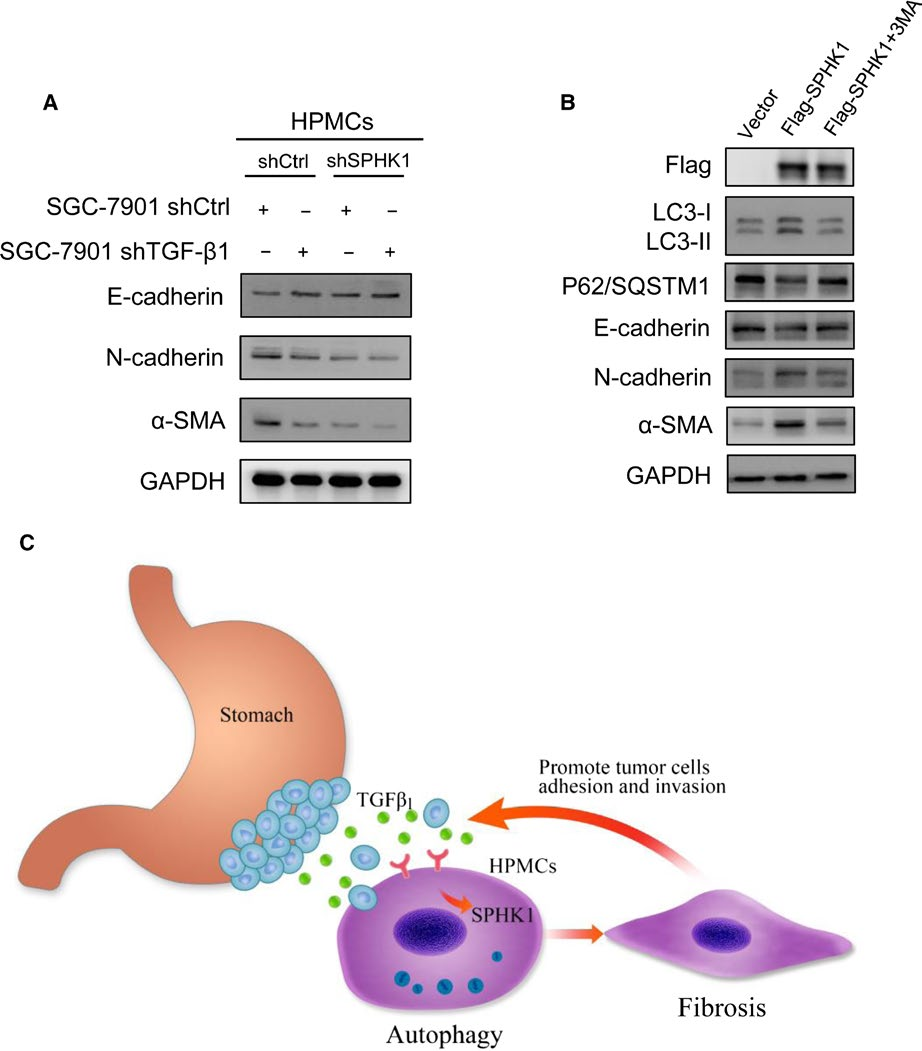
SPHK1 regulated HPMC fibrosis by promoting autophagy. (A) HPMCs were transfected with shCtrl or shSPHK1 and cocultured with SGC‐7901 cells with existence or inhibition of TGF‐β1. Western blotting analyzed MMT‐related proteins E‐cadherin, N‐cadherin, and α‐SMA expression in HPMCs. (B) HPMCs were transfected with vector or Flag‐SPHK1 plasmids and treated with 3‐MA (5 mmol L^−1^). Autophagy‐related proteins and MMT‐related proteins expression was detected by western blot. (C) Schematic diagram of the current study

Previously, we reported that TGF‐β1 induced peritoneal fibrosis and promoted GCPD.^6^, ^7^ Thus, we investigated whether SPHK1 mediated TGF‐β1‐induced peritoneal fibrosis. In the coculture system of GC cells and HPMCs, we detected the expression of epithelial and mesenchymal proteins in HPMCs by western blotting. The increased expression of E‐cadherin and decreased expression of N‐cadherin and α‐SMA suggested that TGF‐β1 downregulation in SGC‐7901 cells significantly weakened TGF‐β1‐induced HPMC fibrosis (Figure 6A). Moreover, the fibroblastic phenotype was substantially attenuated in shSPHK1 HPMCs under TGF‐β1 paracrine action.

To further confirm the effects of SPHK1 on HPMCs autophagy and fibrosis, we overexpressed SPHK1 via Flag‐SPHK1 plasmid transfection. SPHK1 overexpression resulted in increased LC3‐II expression and decreased p62/SQSTM1 expression (Figure 6B). Meanwhile, E‐cadherin downregulation and N‐cadherin and α‐SMA upregulation were also observed in Flag‐SPHK1 HPMCs, indicating that SPHK1 induced fibrosis in HPMCs. Studies have indicated that autophagy is a regulator of fibrogenesis in some tissues and cells.^27^, ^28^ Hence, we explored the regulation of fibrosis by SPHK1‐induced autophagy. We treated SPHK1‐overexpressing HPMCs with 5 mmol L^−1^ 3‐MA. The results demonstrated that autophagy was decreased in HPMCs. Moreover, the upregulation of N‐cadherin and α‐SMA and downregulation of E‐cadherin were reversed. Taken together, these data suggested that SPHK1‐induced autophagy participates in regulating HPMCs fibrogenesis (Figure 6C).

## 4 DISCUSSION

Peritoneal dissemination has been recognized as the most common form of metastasis in advanced GC as well as the leading cause of death in patients with GC.^2^, ^3^ Many efforts have been made in clinical research, but the results are unsatisfactory. The mechanism of GCPD needs further investigation.

The hypothesis of “seed and soil” has been widely accepted, which suggests that the interaction between cancer cells and the peritoneal microenvironment leads to PD.^29^ A completely confluent mesothelial layer is the first barrier against bacterial invasion and tumor attachment.^30^ Our previous results demonstrated that intraperitoneal exfoliated GC cells induced senescence and apoptosis in HPMCs, leading to exposure of the subcutaneous matrix and forming a favorable apterium for GC cell implantation.^22^, ^31^ This injury of HPMCs was mainly mediated by TGF‐β1. Meanwhile, paracrine TGF‐β1 from GC cells induced HPMCs fibrosis, which increased the adhesion of GC cells to the peritoneum and endowed GC cells with enhanced invasiveness.^7^

TGF‐β1 is a member of the cytokine family that has been implicated in cell growth, differentiation, and apoptosis, and it is considered a crucial regulator of fibrosis.^32^ Functionally, TGF‐β1‐mediated regulation of autophagy has recently been reported in physiological and disease conditions.^33^ In the present study, we used GC cell/HPMC coculture systems and found that autophagy was activated in HPMCs by TGF‐β1 released from GC cells. This phenomenon was reduced in the presence of TGF‐β1 receptor inhibitor. Meanwhile, we found that TGF‐β1‐induced HPMCs autophagy promoted GC cell adhesion and invasion.

TGF‐β1 regulates autophagy in different cell types through multiple signaling pathways. It was reported that continuous TGF‐β exposure induced breast cancer cell autophagy and proteolytic degradation of Disabled‐2.^34^ This process was mediated by cathepsin B. In human cardiac fibroblasts, TGF‐β1 promotes fibrogenesis and autophagic activation.^35^ In the present study, SPHK1 was upregulated by TGF‐β1 treatment, and the pivotal role of SPHK1 in TGF‐β1‐induced HPMCs autophagy was confirmed by depleting SPHK1 in HPMCs. However, the precise molecular mechanism by which SPHK1 regulates HPMC autophagy is currently unclear, and we will conduct further investigation in the future.

In the current study, we first discovered that high SPHK1 expression in HPMCs was correlated with LC3B expression, peritoneal recurrence, and poor prognosis in patients with GC. TGF‐β1‐driven HPMCs autophagy promoted the adhesion and invasion of GC cells by regulating SPHK1 in vitro, thus stimulating GCPD in vivo. Our previous studies identified the central role of HPMCs fibrosis in GCPD. Additionally, studies have suggested that SPHK1 is activated in liver and renal tubular fibrosis.^24^, ^36^ Thus, we hypothesized that SPHK1‐mediated autophagy might be a regulator of HPMCs fibrosis. The results illustrated that HPMC fibrosis was substantially reduced in shSPHK1 HPMCs under the paracrine action of TGF‐β1. Consistently, SPHK1 overexpression was associated with increased fibrosis and autophagy. Interestingly, HPMCs fibrosis was reduced in the presence of an autophagy inhibitor. These results suggested that SPHK1‐mediated autophagy instigates TGF‐β1‐induced HPMCs fibrosis, which might be the mechanism by which GCPD is promoted (Figure 6C).

In conclusion, this study demonstrated that TGF‐β1 induces autophagy in HPMCs and promotes GCPD through SPHK1. SPHK1‐mediated autophagy might be a regulator of HPMC fibrosis. Our results provided new data for understanding the mechanisms of GCPD and established SPHK1 as a novel target for controlling GCPD.

## ACKNOWLEDGMENT

This work was supported by National Natural Science Foundation of China (No. 81572334, No.81772549, and No.81602522). We thank Joe Barber Jr., PhD, from Liwen Bianji, Edanz Editing China, for editing the English text of a draft of this manuscript.

## CONFLICT OF INTEREST

The authors declare no conflict of interests.
