# Supplementary figures and images for: SPHK1‐induced autophagy in peritoneal mesothelial cell enhances gastric cancer peritoneal dissemination
Source: Cancer Med. 2019 Feb 21;8(4):1731–43. doi: 10.1002/cam4.2041 (PMC6488120; doi:10.1002/cam4.2041)

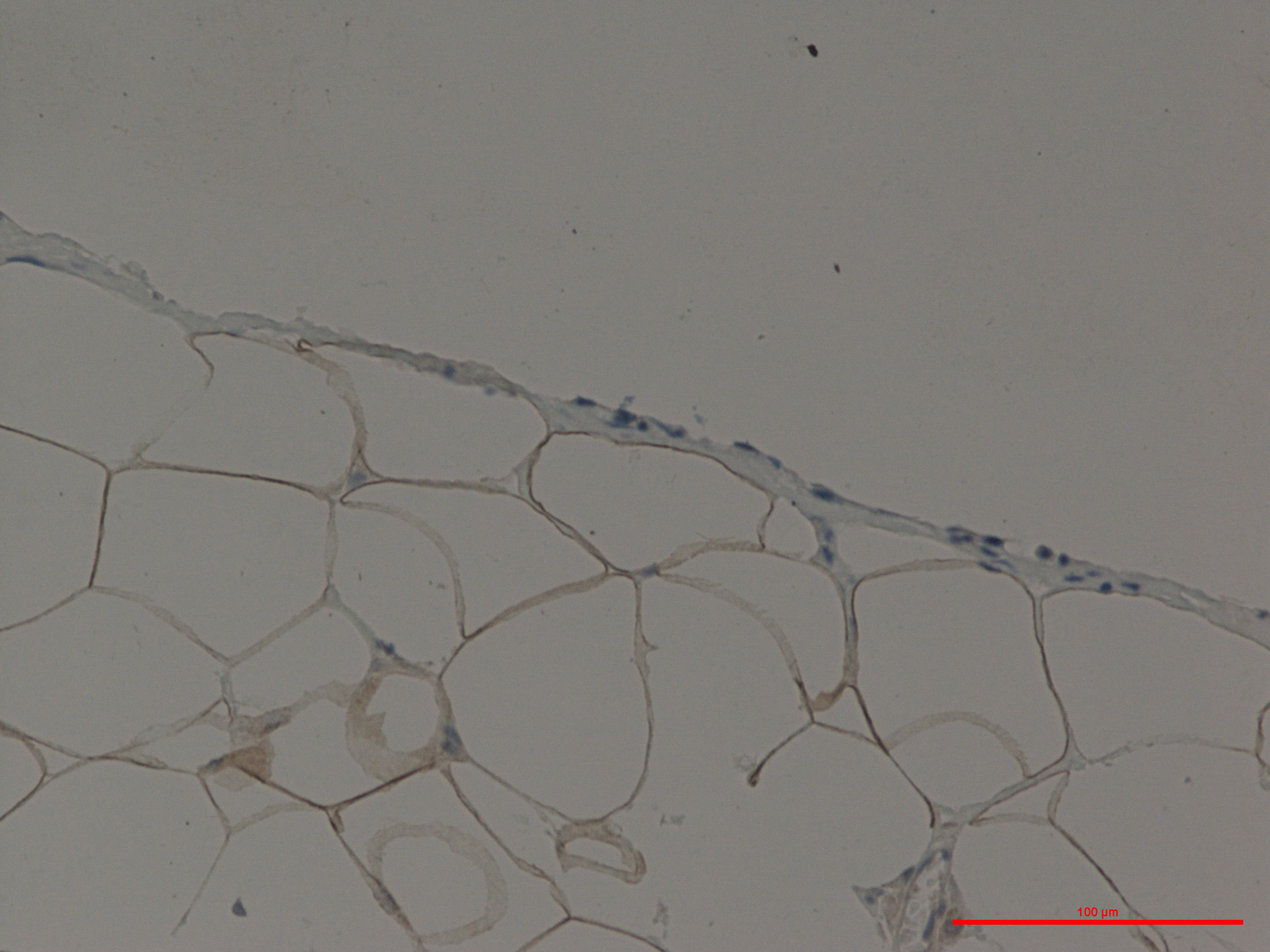

Supplement: Supplementary file 1 [file CAM4-8-1731-s001.tif]

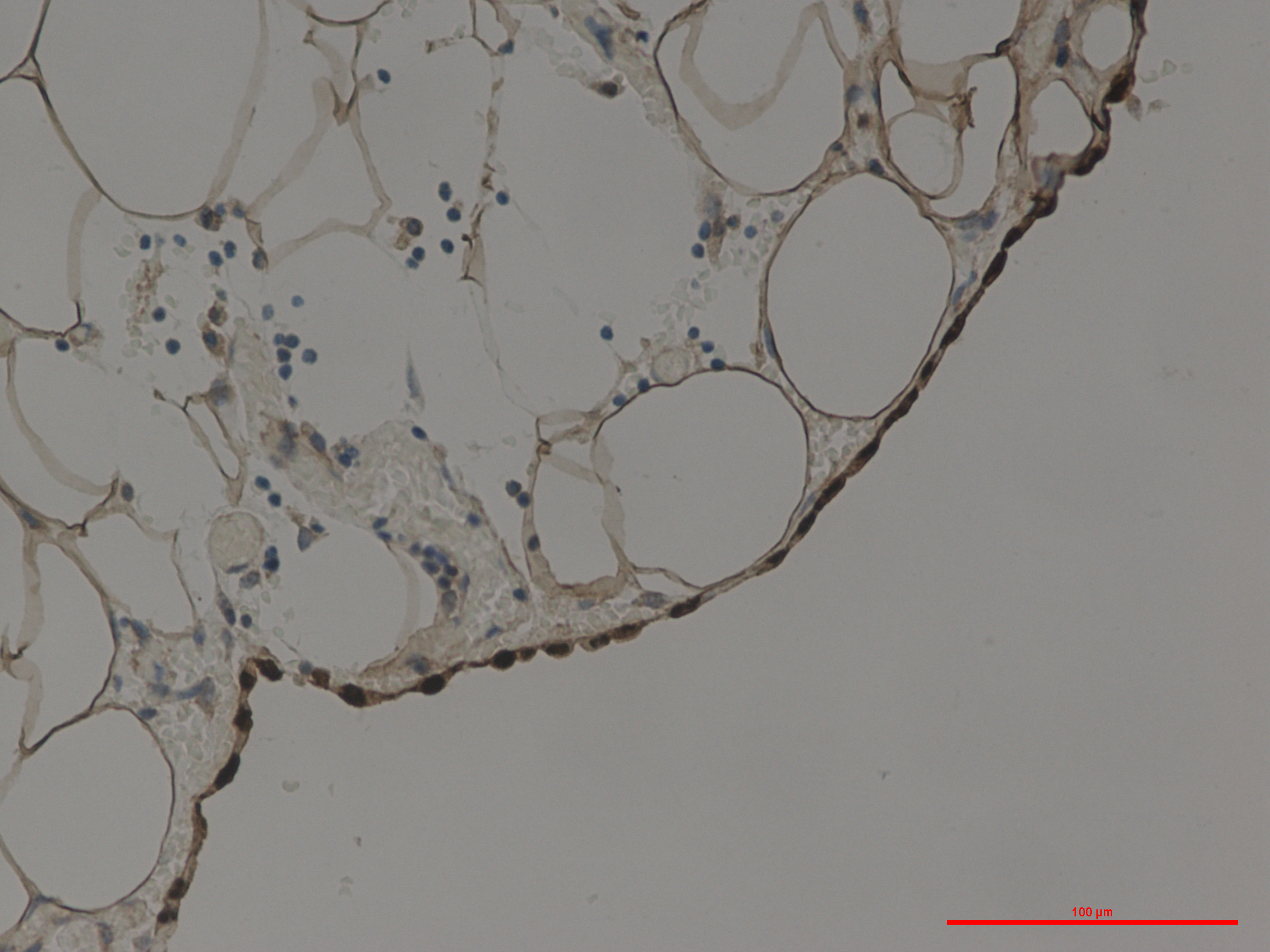

Supplement: Supplementary file 2 [file CAM4-8-1731-s002.tif]

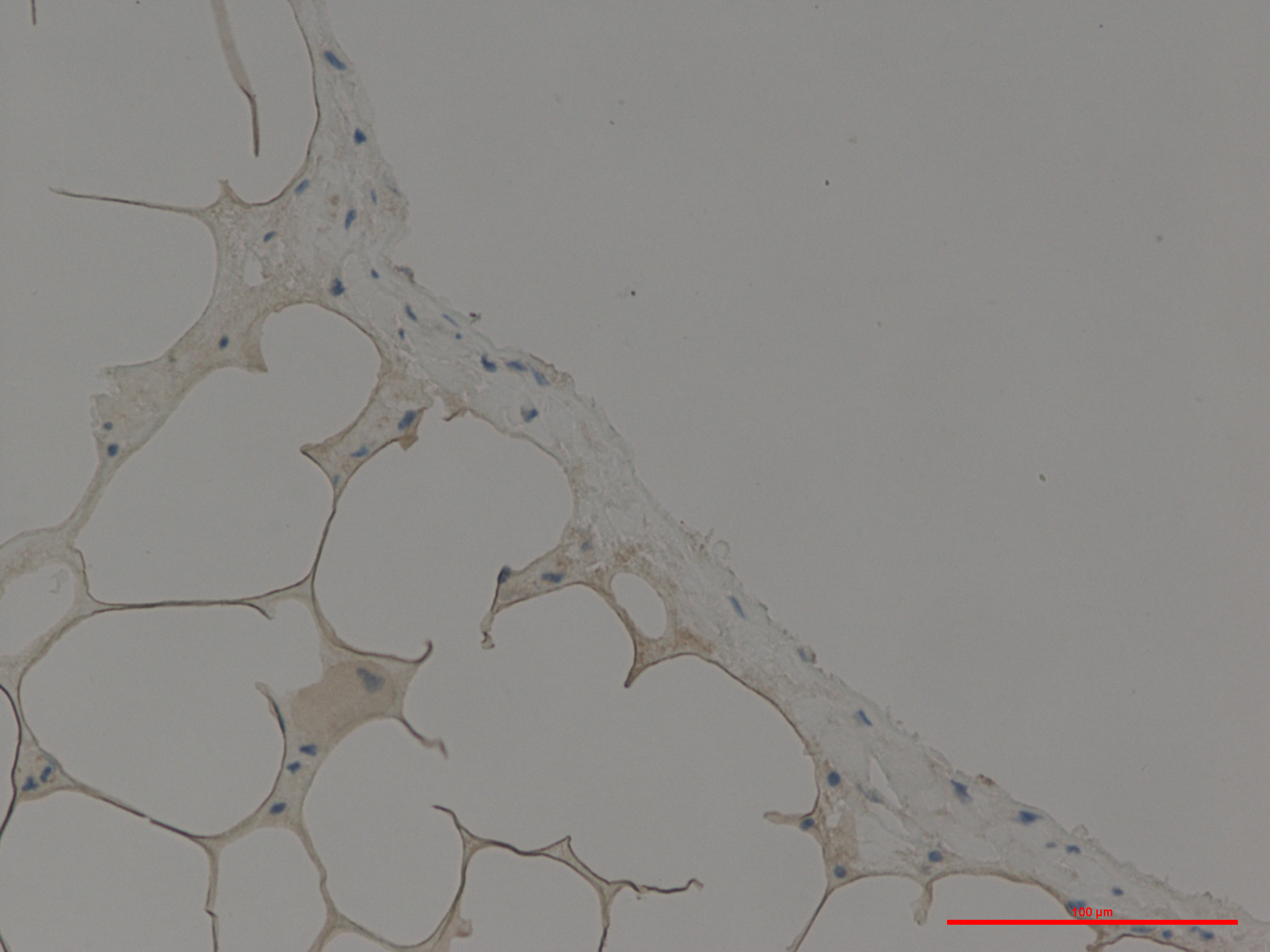

Supplement: Supplementary file 3 [file CAM4-8-1731-s003.tif]

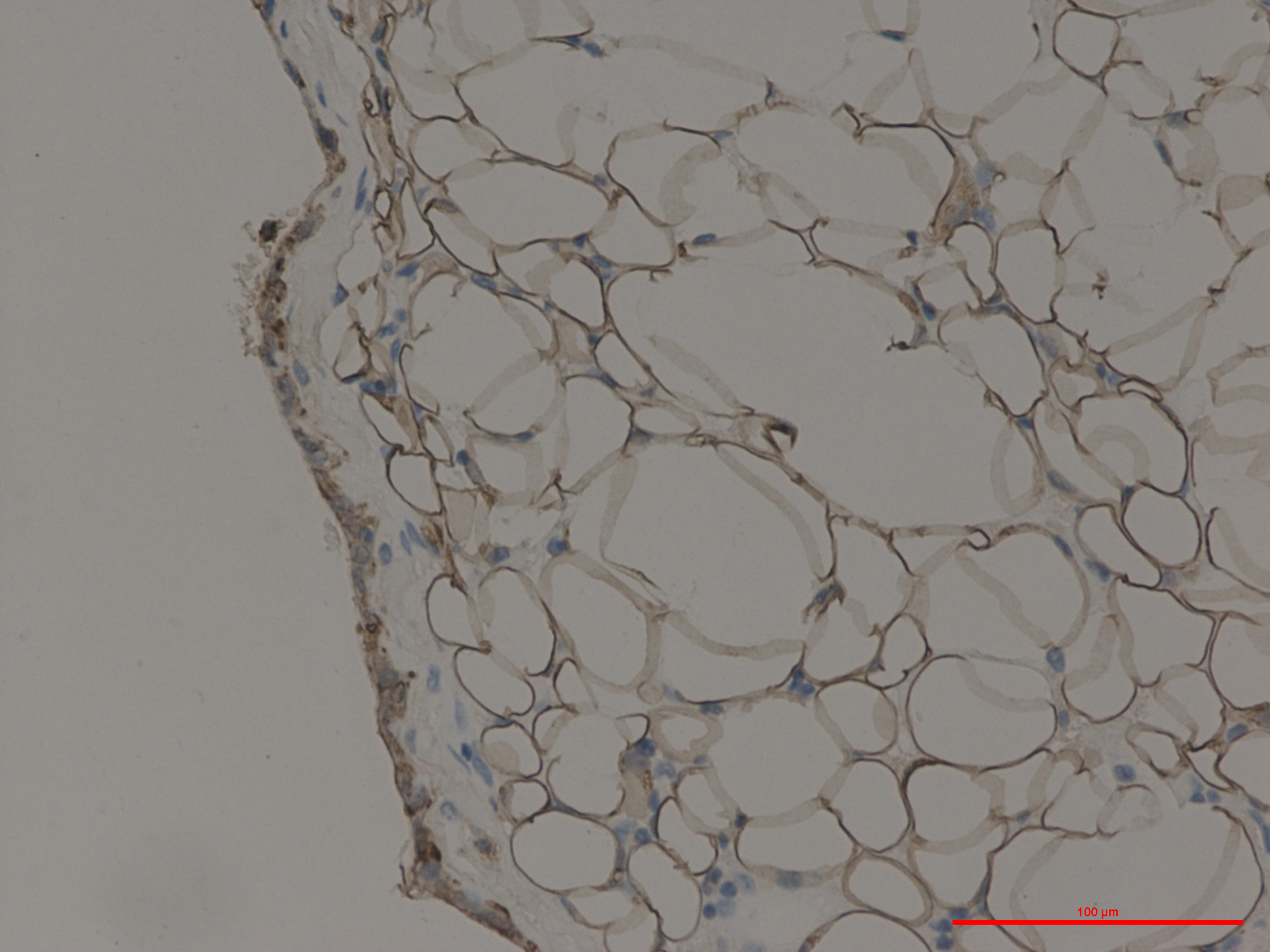

Supplement: Supplementary file 4 [file CAM4-8-1731-s004.tif]
